# A cross-sectional screening by next-generation sequencing reveals *Rickettsia*, *Coxiella*, *Francisella*, *Borrelia*, *Babesia*, *Theileria* and *Hemolivia* species in ticks from Anatolia

**DOI:** 10.1186/s13071-018-3277-7

**Published:** 2019-01-11

**Authors:** Annika Brinkmann, Olcay Hekimoğlu, Ender Dinçer, Peter Hagedorn, Andreas Nitsche, Koray Ergünay

**Affiliations:** 1 Center for Biological Threats and Special Pathogens 1 (ZBS 1), Robert Koch Institute, 13353 Berlin, Germany; 2 Department of Biology, Division of Ecology, Hacettepe University, Faculty of Science, 06800 Ankara, Turkey; 3 Advanced Technology Education, Research and Application Center, Mersin University, 33110 Mersin, Turkey; 4 Department of Medical Microbiology, Virology Unit, Hacettepe University, 06100 Ankara, Turkey

**Keywords:** Tick, Next generation sequencing, *Rickettsia*, *Coxiella*, *Francisella*, *Borrelia*, *Babesia*, *Theilera*, *Hemolivia*, Turkey

## Abstract

**Background:**

Ticks participate as arthropod vectors in the transmission of pathogenic microorganisms to humans. Several tick-borne infections have reemerged, along with newly described agents of unexplored pathogenicity. In an attempt to expand current information on tick-associated bacteria and protozoans, we performed a cross-sectional screening of ticks, using next-generation sequencing. Ticks seeking hosts and infesting domestic animals were collected in four provinces across the Aegean, Mediterranean and Central Anatolia regions of Turkey and analyzed by commonly used procedures and platforms.

**Results:**

Two hundred and eighty ticks comprising 10 species were evaluated in 40 pools. Contigs from tick-associated microorganisms were detected in 22 (55%) questing and 4 feeding (10%) tick pools, with multiple microorganisms identified in 12 pools. *Rickettsia 16S* ribosomal RNA gene, *gltA*, *sca1* and *ompA* sequences were present in 7 pools (17.5%), comprising feeding *Haemaphysalis parva* and questing/hunting *Rhipicephalus bursa*, *Rhipicephalus sanguineus* (*sensu lato*) and *Hyalomma marginatum* specimens. A near-complete genome and conjugative plasmid of a *Rickettsia hoogstraalii* strain could be characterized in questing *Ha. parva*. *Coxiella*-like endosymbionts were identified in pools of questing (12/40) as well as feeding (4/40) ticks of the genera *Rhipicephalus*, *Haemaphysalis* and *Hyalomma*. *Francisella*-like endosymbionts were also detected in 22.5% (9/40) of the pools that comprise hunting *Hyalomma* ticks in 8 pools. *Coxiella*-like and *Francisella*-like endosymbionts formed phylogenetically distinct clusters associated with their tick hosts. *Borrelia turcica* was characterized in 5% (2/40) of the pools, comprising hunting *Hyalomma aegyptium* ticks. Co-infection of *Coxiella*-like endosymbiont and *Babesia* was noted in a questing *R. sanguineus* (*s.l.*) specimen. Furthermore, protozoan *18S* rRNA gene sequences were detected in 4 pools of questing/hunting ticks (10%) and identified as *Babesia ovis*, *Hemolivia mauritanica*, *Babesia* and *Theileria* spp.

**Conclusions:**

Our metagenomic approach enabled identification of diverse pathogenic and non-pathogenic microorganisms in questing and feeding ticks in Anatolia.

**Electronic supplementary material:**

The online version of this article (10.1186/s13071-018-3277-7) contains supplementary material, which is available to authorized users.

## Background

Ticks (class Arachnida, subclass Acari) are the most significant arthropod vectors, along with mosquitoes, participating in the transmission of pathogens to humans [1]. A diverse group of infectious agents including viruses, bacteria and protozoans can be transmitted by ticks, surpassing most arthropods in terms of vector potential [2]. Tick-borne infections of humans are of zoonotic origin, with pathogens maintained in natural cycles involving tick vectors and animal hosts [3]. Frequently, humans are accidental, dead-end hosts that do not significantly contribute to the pathogen’s life-cycle. Various tick species occupy distinct ecological niches that define their distribution patterns and risk areas for tick-borne infections [4]. The past decades have witnessed the emergence and resurgence of several tick-borne infections with considerable impact on human and animal welfare [1, 5]. A deeper understanding of the epidemiology and potential public health threats of tick-borne infections rely on effective surveillance programmes to identify circulating pathogens in vectors and reliable diagnosis of vertebrate infections.

In addition to the tick-borne pathogens, a diverse group of commensal and symbiotic bacteria are described in ticks, usually co-circulating with the infectious agents [6]. Their biology and effect on tick life-cycle remain largely unexplored, despite evidence suggesting their involvement in fitness, nutritional adaptation, defense and immunity [7]. These microorganisms are also likely to interact with the replication and transmission of tick-borne pathogens, with potential implications for human and animal health [7, 8].

Turkey is located in Asia Minor and maintains a natural transmission zone for vector-borne infections between Asia, Africa and Europe [9]. The geographical regions of Anatolia, with diverse climate conditions, vegetation patterns, domestic animals and wildlife provide suitable habitats for perpetuating several arthropod vectors of disease, including ticks [9]. Several species of the families Ixodidae and Argasidae are present in the tick fauna of Turkey [10]. Human tick-borne infections have also been documented, caused by protozoans, nematodes, bacteria and viruses [9, 11]. We have recently reported the presence of several RNA viruses in ticks collected from various regions of Anatolia [12]. In the present study, we aimed to perform a cross-sectional screening by using next-generation sequencing (NGS) to characterize tick-associated bacteria and protozoans.

## Methods

### Specimen collection and processing

Ticks collected in several locations from Ankara and Cankiri provinces (central Anatolia), Mugla Province (western Anatolia, Aegean region) and Mersin Province (southern Anatolia, Mediterranean region) from April to October from 2014 to 2016 were evaluated. Questing ticks were captured on site by flagging as well as from infested domesticated animals: dogs (*Canis familiaris*); cattle (*Bos taurus*); and goats (*Capra aegagrus hircus*). The ticks were kept alive individually in vials, transferred to the laboratory and identified morphologically to the species level using several taxonomic keys [13–17]. Following identification, the specimens were pooled according to species and collection site up to a maximum of 22 individuals per pool and stored at -80 °C for further analysis.

Individual and pooled ticks with up to five specimens were homogenized using the SpeedMill PLUS (Analytik Jena, Jena, Germany), and total nucleic acid purification was performed by using BlackPREP tick DNA/RNA kit (Analytik Jena) according to the manufacturer’s instructions. Pools with six or more specimens were kept in 500–700 μl of Eagle’s minimal essential medium, supplemented with 1% L-glutamine and 5% fetal bovine serum. These pools were homogenized by vortexing with tungsten carbide beads (Qiagen, Hilden, Germany) and clarified by centrifugation for 4 min at 4000× *rpm*. Subsequently, the ground pools were aliquoted and subjected to nucleic acid extraction using High Pure Viral Nucleic Acid Kit (Roche Diagnostics, Mannheim, Germany)

### Next-generation sequencing (NGS) and phylogenetic analysis

Purified nucleic acids from tick pools were reverse transcribed with random hexamer primers to double-stranded cDNA using SuperScript IV Reverse Transcriptase (Thermo Fisher Scientific, Hennigsdorf, Germany) and NEBNext mRNA Second Strand Synthesis Module (New England Biolabs, Frankfurt am Main, Germany). Agencourt AMPure XP Reagent (Beckman Coulter Biosciences, Krefeld, Germany) and Agilent 2100 Bioanalyzer (Agilent Technologies, Waldbronn, Germany) were employed for cleanup, yield and size distribution determination. Fragmentation, adaptor ligation and amplification were carried out using NexteraXT DNA Library Preparation Kit (Illumina Inc., San Diego, CA, USA) according to the manufacturer’s protocols. Sequencing runs were performed on an Illumina HiSeq (Illumina Inc.) instrument in paired-end mode.

The raw sequencing data was de-multiplexed and extracted in fastq format. Trimmomatic software was employed for trimming for quality and length with a phred score of 33 and a minimum length of 30 base pairs (bp) and removal of Illumina adaptors [18]. Obtained reads were aligned to the GenBank RefSeq databases of the National Center for Biotechnology Information (NCBI) for bacteria (v.17.03.2017), *16S* ribosomal RNA (rRNA) (RefSeq rRNA, v.01.08.2017) and selected protozoa (in-house curated database, sequences available upon request, v.29.09.2017) using MALT (MEGAN alignment tool, v0.3.8) and MEGAN (Metagenome Analyzer, v. 6.12.3) [19, 20]. Aligned reads were extracted and assembled into contigs using Velvet (v.1.2.10) with a k-mer length of 31 [21]. The contigs were checked for heterogeneity by visual inspection and *via* pairwise identity values using Geneious software v.11.1.5 (Biomatters Ltd, Auckland, New Zealand). The *16S* rRNA gene sequences were scanned for chimeras with divergence of > 3% from the closest parent using UCHIME2, implemented at the NCBI database [22]. For the near-complete genome and plasmid sequences, contigs and remaining reads were mapped to closely related strains. BLASTn, BLASTn optimized for highly similar sequences (MEGABLAST) and BLASTp algorithms were used for nucleotide and deduced amino acid similarity searches in the public databases implemented in the NCBI website (www.ncbi.nlm.nih.gov/blast/) [23]. Nucleotide and putative amino acid alignments and pairwise sequence comparisons were generated by using the CLUSTAL W program implemented within Geneious software [24]. Conserved protein domain and motif searches were performed using the web search tool (https://www.ncbi.nlm.nih.gov/Structure/cdd/wrpsb.cgi) and MOTIF Search (http://www.genome.jp/tools/motif/) in the PFAM database [25, 26]. The models for the phylogenetic and molecular evolutionary analyses were selected using the best-fit DNA/protein-substitution model tools of the MEGA v.6.06 software [27]. Phylogenetic trees were constructed using the maximum-likelihood method with the Tamura-Nei substitution model. The reliability of the inferred trees was evaluated by bootstrap analysis of 1000 replicates.

## Results

Two-hundred eighty ticks, comprising 179 female (63.9%), 100 male (35.7%) and 1 nymph (0.4%) specimens were evaluated in 40 pools, prepared according to species and collection site (Additional file 1: Table S1). A total of 10 tick species were identified among which *Rhipicephalus bursa* (*n* = 76; 27.1%), *Hyalomma aegyptium* (*n* = 49; 17.5%) and *Haemaphysalis parva* (*n* = 46; 16.4%) represented the most abundant species. A total of 8 pools with *Ha. parva* (*n* = 3), *Rhipicephalus sanguineus* (*s.l.*) (*n* = 3), *Dermacentor marginatus* (*n* = 1) and *Rhipicephalus annulatus* (*n* = 1) specimens were collected from animal hosts, whereas the remaining pools (*n* = 32, 80%) comprised questing/hunting ticks (Additional file 1: Table S1).

NGS provided trimmed read numbers of 67,753–53,026,910 (mean = 5,061,800; median = 2,430,793) in the tick pools (Additional file 1: Table S1). Tick-associated microbial sequences were detected in 26/40 pools (65%), with multiple microorganisms identified in 12 (46.2%) of these positive pools. *Coxiella*, *Francisella*, *Rickettsia*, *Babesia*, *Borrelia*, *Theileria* and *Hemolivia* sequences were characterized in reactive tick pools. *Coxiella* spp. were the most frequently detected microorganism, identified in 16 of the 40 pools (40%), followed by *Francisella* spp. (22.5%), *Rickettsia* spp. (17.5%) and other microorganisms (Table 1, Additional file 1: Table S2).

**Table 1:** Tick pools with detectable microorganism sequences

Pool code	Source	Species	Microorganism						
			*Coxiella*	*Francisella*	*Rickettsia*	*Borrelia*	*Babesia*	*Theilera*	*Hemolivia*
P3	Animal host	*R. sanguineus* (*s.l.*)	+	-	-	-	-	-	-
P11	Questing	*R. sanguineus* (*s.l.*)	-	+	-	-	-	+	-
P16	Questing	*R. sanguineus* (*s.l.*)	+	-	-	-	-	-	-
P19	Questing	*R. sanguineus* (*s.l.*)	+	-	-	-	-	-	-
P21	Questing	*R. sanguineus* (*s.l.*)	+	-	+	-	-	-	-
P23	Questing	*R. sanguineus* (*s.l.*)	+	-	-	-	+	-	-
P14	Questing	*R. bursa*	+	-	-	-	-	-	-
P15	Questing	*R. bursa*	+	-	-	-	-	-	-
P18	Questing	*R. bursa*	+	-	+	-	-	-	-
P24	Questing	*R. bursa*	+	-	-	-	-	-	-
P34	Questing	*R. bursa*	-	-	-	-	+	-	-
P28	Questing	*Rhipicephalus* spp.	+	-	-	-	-	-	-
P12	Hunting	*H. aegyptium*	-	-	-	+	-	-	-
P20	Hunting	*H. aegyptium*	-	+	-	-	-	-	-
P22	Hunting	*H. aegyptium*	-	+	-	-	-	-	-
P25	Hunting	*H. aegyptium*	-	+	-	+	-	-	-
P26	Hunting	*H. aegyptium*	-	+	-	-	-	-	+
P35	Hunting	*H. marginatum*	-	+	+	-	-	-	-
P37	Hunting	*H. marginatum*	-	+	+	-	-	-	-
P38	Hunting	*H. marginatum*	-	+	-	-	-	-	-
P40	Hunting	*H. marginatum*	+	+	-	-	-	-	-
P13	Hunting	*H. excavatum*	+	-	-	-	-	-	-
P4	Animal host	*Ha. parva*	+	-	+	-	-	-	-
P5	Animal host	*Ha. parva*	+	-	+	-	-	-	-
P6	Animal host	*Ha. parva*	+	-	+	-	-	-	-
P39	Questing	*D. marginatus*	+	-	-	-	-	-	-
Total			16	9	7	2	2	1	1

*Key*: +, detected; -, not detected

### *Rickettsia* findings

*Rickettsia* spp. sequences were identified in a total of 7 tick pools comprising *Ha. parva* (*n* = 3), *Hyalomma marginatum* (*n* = 2), *R. bursa* (*n* = 1) and *R. sanguineus* (*s.l.*) (*n* = 1) specimens (Table 2). *Rickettsia* spp. were detected in 4 pools (57.1%) of questing/hunting and 3 pools (42.9%) of feeding ticks.

**Table 2:** Tick pools with *Rickettsia* spp. contigs. Size and GenBank accession numbers are provided

Pool	Target gene						Identification
	*16S* rRNA	*23S* rRNA	*ompA*	*sca1*	*gltA*	Plasmid	
P4^a^	1508 bp (MH645181)	2761 bp (MH618686)	830 bp (MH630146)	1028 bp (MH630145)	1308 bp (MH630144)	2376 bp (MH649269)	*R. hoogstraalii*
P5	1395 bp (MH645180)	–	–	–	355 bp (MH673723)	–	*Rickettsia* sp.
P6	1426 bp (MH645175)	–	1133 bp (MH649268)	428 bp (MH630147)	821 bp (MH673722)	–	*R. hoogstraalii*
P18	1232 bp (MH645179)	–	–	–	–	–	*Rickettsia* sp.
P21	1309 bp (MH645178)	–	–	–	–	–	*Rickettsia* sp.
P35	1433 bp (MH645176)	–	–	–	–	–	*Rickettsia* sp.
P37	1392 bp (MH645177)	–	–	–	–	–	*Rickettsia* sp.

^a^Sequences obtained from the near-complete genome (bp: base pairs)

A near-complete *Rickettsia* genome was assembled from the pool P4 that comprised 13 feeding *Ha. parva* ticks. A total of 82,002 reads from this pool were aligned to the genomes of two *Rickettsia* strains, *R. hoogstraalii* strain Croatica and *Rickettsia felis* strain URRWXCal2 (CP000053). These were further assembled into 1516 contigs with an N50 length of 1012 bp and a total length of 1,176,263 bp. Pairwise comparison of this sequence revealed 98.3 and 89.6% identity with *R. hoogstraalii* and *R. felis*, respectively. The sequence was disrupted by several gaps of varying length and further sequencing to complete the genome was not feasible. Therefore, we extracted intact contigs for comparison, including complete *16S* and *23S* rRNA genes, citrate synthase (*gltA*), surface cell antigen 1 (*sca1*) and outer membrane protein A (*ompA*). These sequences were submitted to the GenBank database (Table 2) and the assembled genome sequence is available in FASTA format as Additional file 2.

A 2376 bp section of the *Rickettsia* putative conjugative plasmid was also detected in pool P4. Pairwise comparison showed 80.8 and 97.8% identity with *R. australis* and *R. hoogstraalii* plasmids, respectively. A comparative alignment is provided in Additional file 3. Motifs of TraA_Ti conjugative transfer protein, MobA/MobL family mobilization protein and TraA conjugal transfer relaxase were identified within the sequence.

**Fig. 1:**
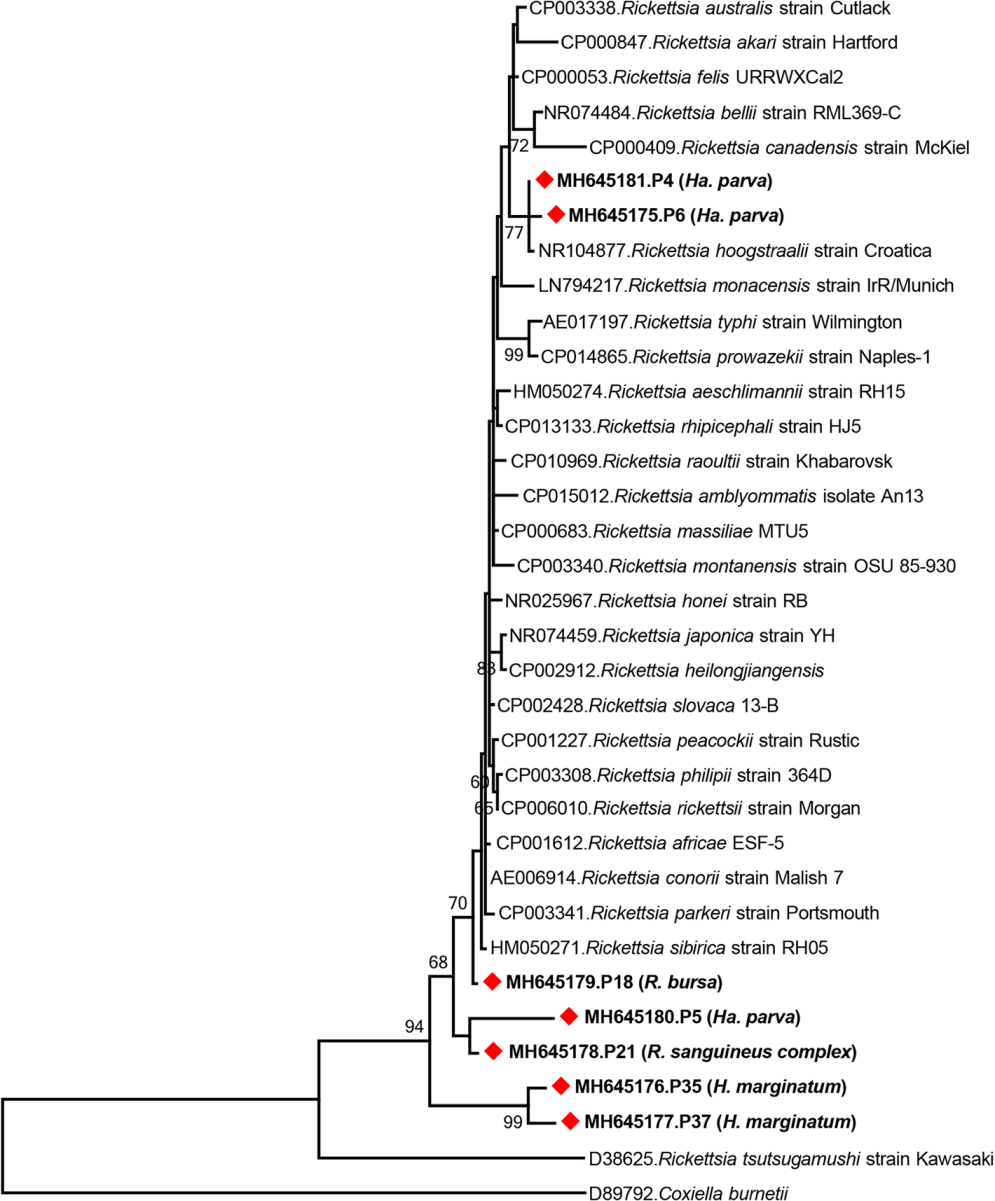
Maximum-likelihood analysis of the *Rickettsia* partial *16S* rRNA gene sequences (1294 nucleotides). The tree was constructed using the Tamura-Nei model, with a bootstrap analysis of 1000 replicates. Sequences characterized in this study are given in bold and indicated with a symbol, GenBank accession number, pool code and host tick species. *Rickettsia* strains are indicated by GenBank accession number, microorganism and strain/isolate name. Bootstrap values lower than 60 are not shown. *Coxiella burnetii* was included as the outgroup

In addition to the complete *16S* rRNA gene sequence in pool 4, *16S* rRNA gene contigs of 1232–1433 bp were obtained in feeding *Ha. parva* pools (P5 and P6), hunting *H. marginatum* pools (P35 and P37), a questing *R. bursa* pool (P18) and a questing *R. sanguineus* (*s.l.*) pool (P21) (Table 2). Contigs in pools P5 and P6 revealed 97–98% identity with *R. hoogstraalii* in BLASTn and MEGABLAST searches. In the maximum-likelihood tree, the P4 and P6 contigs grouped with *R. hoogstraalii*, with separate clustering of P18, P5-P21 and P35–P37 (Fig. 1).

The complete *gltA*-coding region extracted from the *Rickettsia* genome in pool P4 demonstrated 99 and 100% identities to *R. hoogstraalii* prototype isolate, in nucleotide and deduced amino acid comparisons, respectively. The *gltA*-coding sequences were also obtained in pools P5 and P6. These constituted 355 and 821 bp stretches which were identical to the P4 sequence. The *sca1* and *ompA* contigs in pool P4 were also highly similar to *R. hoogstraalii*, with 98.4–99.8% and 99.4% nucleotide and amino acid identity, respectively. The 428-nucleotide *sca1* contig from the pool P6 was also identical to the sequence in pool P4. In addition, a longer section of *ompA* could be obtained from P6, which showed 1.4% divergence from P4 sequence and 98.6 and 99.2% nucleotide and amino acid identity, respectively, to *R. hoogstraalii*.

Overall, the obtained sequences enabled identification of the *Rickettsia* strain in pools P4 and P6 (Table 2), and the analysis of the *16S* region could not provide data sufficient for strain discrimination in pools P5, P18, P21, P37 and P37 (Fig. 1). The available 355-nucleotide *gltA* contig from pool P5 revealed similar identity to several *Rickettsia* in BLASTn and MEGABLAST searches, therefore the precise identification of the strain in this pool also remained obscure.

### *Coxiella*, *Francisella* and *Borrelia* findings

**Fig. 2:**
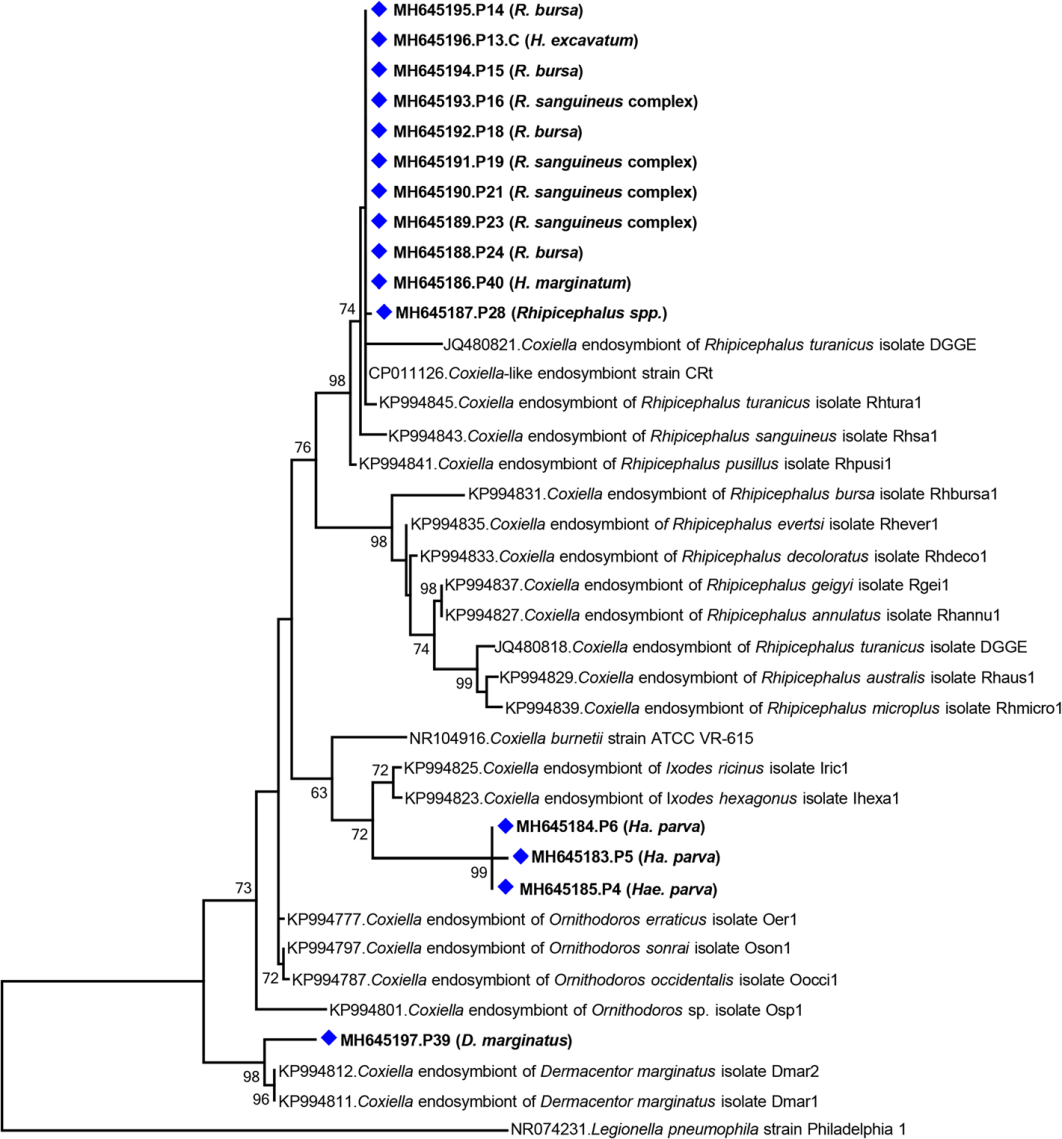
Maximum-likelihood analysis of the *Coxiella* partial *16S* rRNA gene sequences (1086 nucleotides). The tree was constructed using the Tamura-Nei model, with a bootstrap analysis of 1000 replicates. Sequences characterized in this study are given in bold and indicated with a symbol, GenBank accession number, pool code and host tick species. Bacterial strains are indicated by GenBank accession number, microorganism and strain/isolate name. Bootstrap values lower than 60 are not shown. *Legionella pneumophila* strain Philadelphia 1 was included as the outgroup

Bacterial *16S* rRNA gene sequences other than *Rickettsia* were characterized in 26 pools (65%), 22 (84.6%) of which included questing/hunting ticks. In 16 tick pools (40%), comprising *Rhipicephalus*, *Hyalomma*, *Haemaphysalis* and *Dermacentor* specimens, *16S* rRNA gene sequences with varying similarities to several *Coxiella*-like endosymbionts (CLE) were detected. CLEs were present in pools of questing (*n* = 12) as well as feeding (*n* = 4) ticks (Table 1). The sequences comprised 1088–1180 bp with up to 4.5% diversity. In the maximum-likelihood tree, three distinct clusters were observed (Fig. 2). The sequences from *Rhipicephalus* and *Hyalomma* species (MH645186–96) grouped with endosymbionts of *Rhipicephalus* spp., while the sequences from feeding *Ha. parva* (MH645183–5) remained distinct, sharing a common ancestor with CLE from *Ixodes* spp. The sequence originating from the questing *D. marginatus* tick pool (MH645197) also formed another clade with endosymbionts of the same tick species (Fig. 2).

**Fig. 3:**
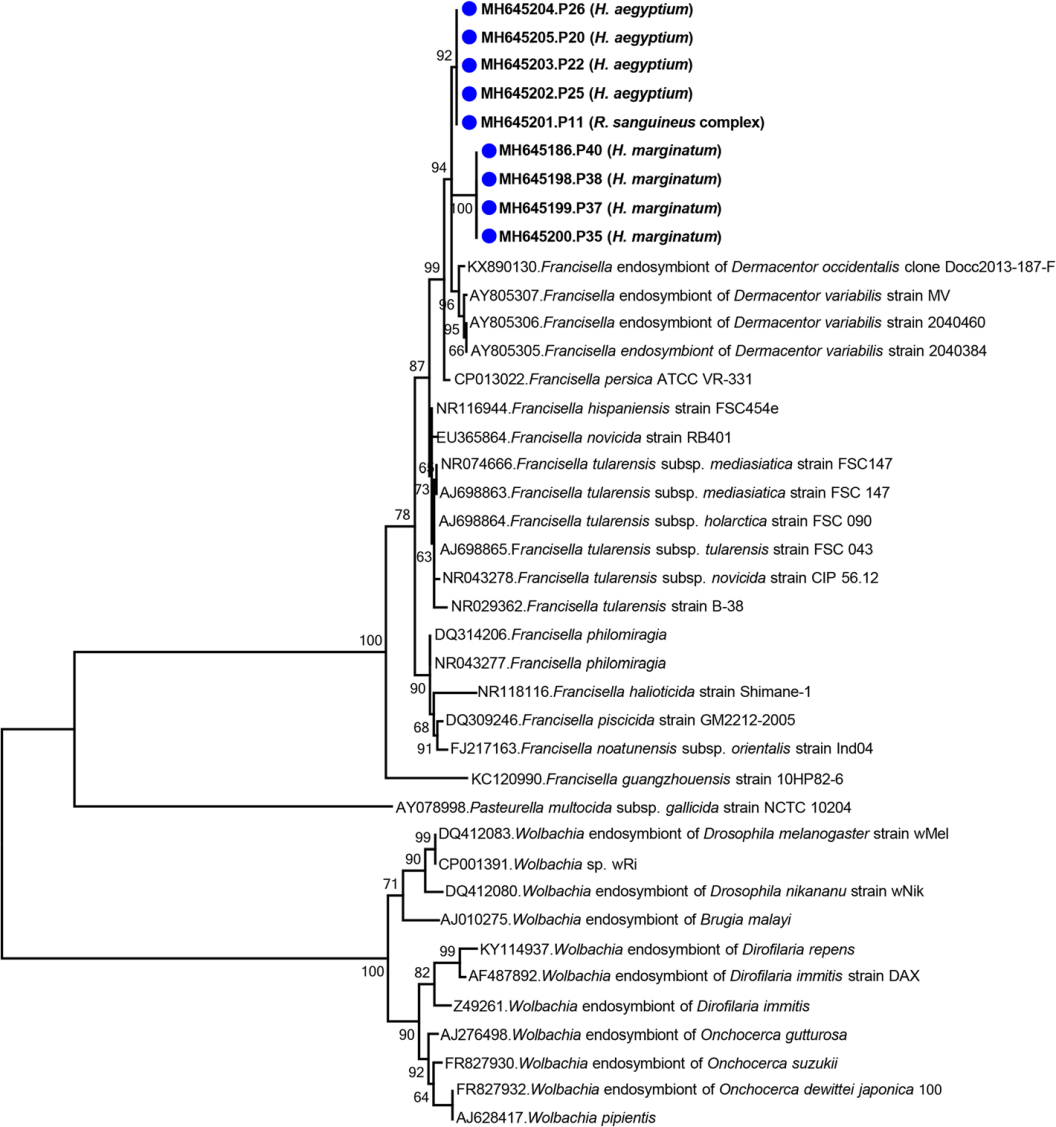
Maximum-likelihood analysis of the *Francisella* and *Wolbachia* partial *16S* rRNA gene sequences (375 nucleotides). The tree was constructed using the Tamura-Nei model, with a bootstrap analysis of 1000 replicates. Sequences characterized in this study are given in bold and indicated with a symbol, GenBank accession number, pool code and host tick species. Bacterial strains are indicated by GenBank accession number, microorganism and strain/isolate name. Bootstrap values lower than 60 are not shown. *Pasteurella multocida* subsp. *gallicida* strain NCTC 10204 was included as the outgroup

The *16S* rRNA gene sequences related to *Francisella* species were identified in 9 pools (22.5%) with hunting/questing specimens. In contrast to CLE, these sequences belonged to more abundant *Hyalomma* ticks (8/9) (Table 1). They comprised contigs of 1290–1516 bp and formed two groups, namely P11, P20, P22, P25, P26 and P35, P37, P38, P40, with less than 1% intragroup divergence and 98.9% identity between groups. These groups, distinct from pathogenic *Francisella* and *Wolbachia* endosymbionts, could also be distinguished phylogenetically, as the pools with *H. marginatum* ticks (MH645186, MH645198-MH645200) formed high bootstrap supported clades with *Francisella*-like endosymbionts (FLE) of *Hyalomma rufipes* (Fig. 3). The other group, detected in *H. aegyptium* and *R. sanguineus* (*s.l.*) (MH645201-MH645205), clustered with sequences from *H. aegyptium* and *Amblyomma* spp., whereas FLE from *Ixodes*, *Dermacentor* and *Haemaphysalis* ticks remained distinct.

**Fig. 4:**
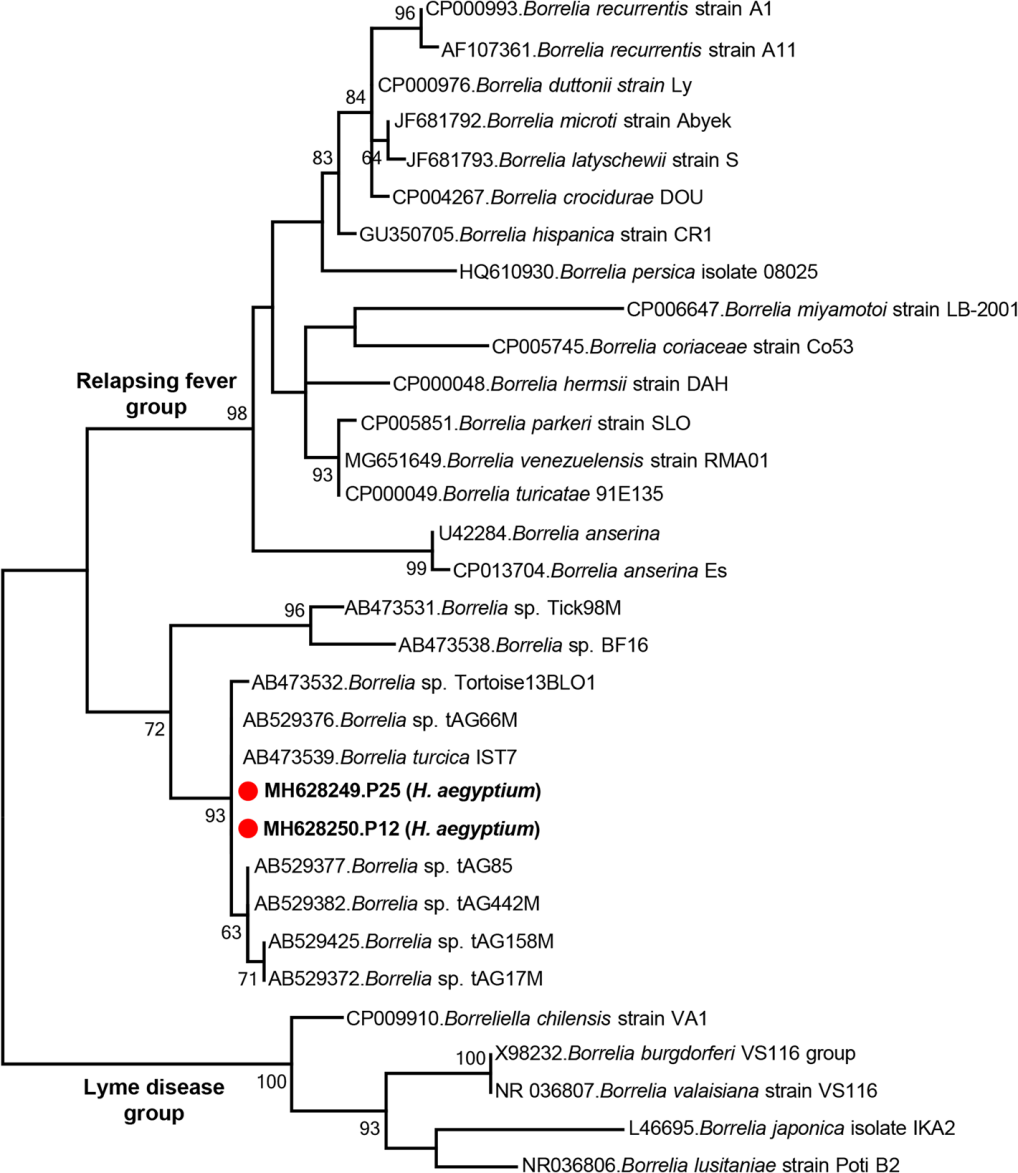
Maximum-likelihood analysis of the *Borrelia* partial *16S* rRNA gene sequences (1352 nucleotides). The tree was constructed using the Tamura-Nei model, with a bootstrap analysis of 1000 replicates. Sequences characterized in this study are given in bold and indicated with a symbol, GenBank accession number, pool code and host tick species. Bacterial strains are indicated by GenBank accession number, microorganism and strain/isolate name. Bootstrap values lower than 60 are not shown

The last group of *16S* rRNA gene contigs constituted two sequences of 1361 and 1364 bp (MH628249-MH628250), identical except for 1–3 nucleotide terminal overhangs. They were detected in pools of hunting *H. aegyptium* ticks and were identical to *B. turcica* and *Borrelia* sp. recovered from *H. aegyptium* in Turkey and *Amblyomma geoemydae* in Japan. They grouped together along with several tick-associated *Borrelia* species in the maximum-likelihood tree, forming a separate clade distinct from relapsing fever and Lyme disease *Borrelia* (Fig. 4).

### *Babesia*, *Theilera* and *Hemolivia* findings

**Fig. 5:**
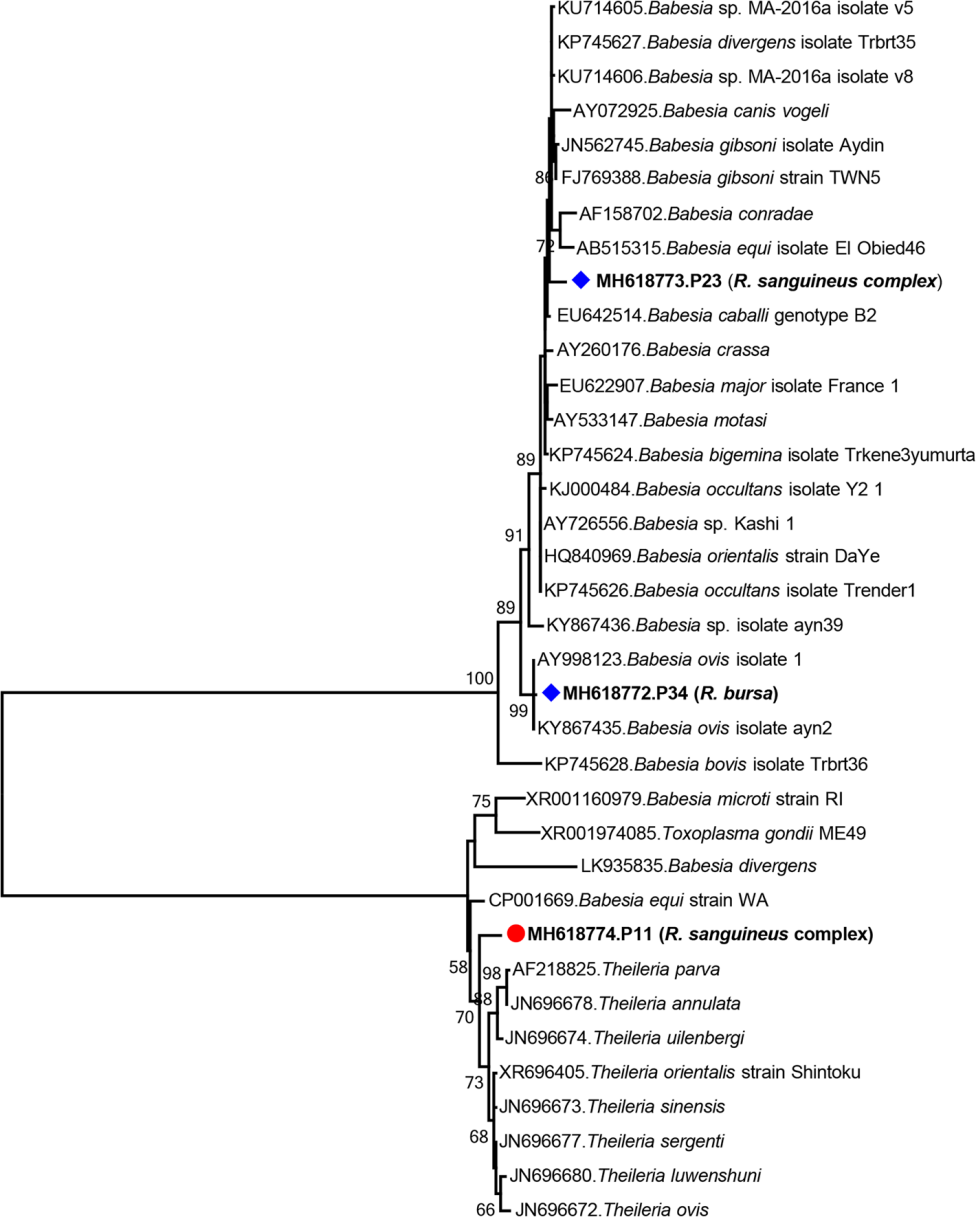
Maximum-likelihood analysis of the *Babesia* and *Theilera* partial *18S* rRNA gene sequences (794 nucleotides). The tree was constructed using the Tamura-Nei model, with a bootstrap analysis of 1000 replicates. Sequences characterized in this study are given in bold and indicated with a symbol, GenBank accession number, pool code and host tick species. Protozoan strains are indicated by GenBank accession number, microorganism and strain/isolate name. Bootstrap values lower than 60 are not shown

Eukaryotic *18S* rRNA gene contigs were obtained in four questing tick pools (10%). In three pools comprising *Rhipicephalus* spp., sequences related to *Babesia* and *Theileria* were detected. BLASTn analysis of the longest sequence (1454 bp) in the pool P34 (MH618772) revealed highest similarity rates of 99% to *B. ovis*. It further grouped phylogenetically with *B. ovis* with high bootstrap values, confirming the identification (Fig. 5). The 544-bp sequence in pool P23 (MH618773) displayed 97–98% identity to several *Babesia* sp. detected in ticks, but no definitive strain identification could be established. The recently described, presumably novel *Babesia* sequences from ticks and goats from Turkey [28, 29] also revealed 96.1–97% identity and were distantly related to this sequence (Fig. 5). The 1339-bp sequence obtained from pool P11 (MH618774) showed 88–89% identity to various *Babesia* and *Theileria* spp. and clustered with the *Theileria* spp. in the maximum-likelihood analysis (Fig. 5).

**Fig. 6:**
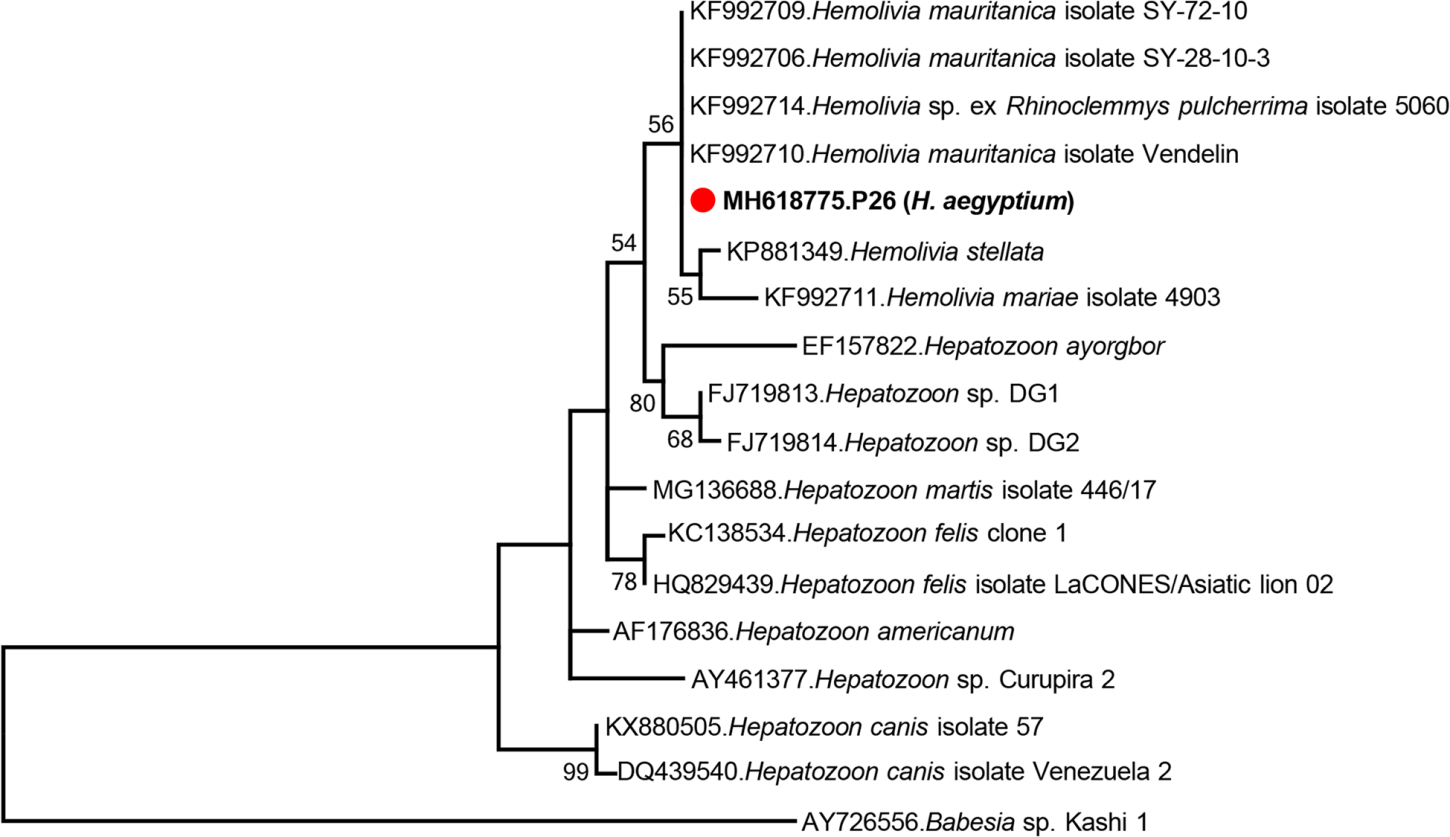
Maximum-likelihood analysis of the *Hemolivia* and *Hepatozoon* partial *18S* rRNA gene sequences (465 nucleotides). The tree was constructed using the Tamura-Nei model, with a bootstrap analysis of 1000 replicates. Sequences characterized in this study are given in bold and indicated with a symbol, GenBank accession number, pool code and host tick species. Protozoan strains are indicated by GenBank accession number, microorganism and strain/isolate name. Bootstrap values lower than 50 are not shown. *Babesia* sp. isolate Kashi1 was included as the outgroup

Finally, a 472 bp sequence, with 99–100% identity to several *Hemolivia mauritanica* isolates, was obtained from a pool of hunting *H. aegyptium* ticks (Table 1). Despite the availability of a relatively short segment, the sequence (MH618775) grouped with *He. mauritanica* isolates in the maximum-likelihood tree (Fig. 6).

## Discussion

We performed a cross-sectional screening for tick-associated bacteria and protozoans, using an NGS-based strategy in pools of field-collected ticks from various regions of Turkey. We adopted a straightforward approach for NGS, using standard and widely-used commercial assays for nucleic acid purification, cDNA and sequencing library preparation, performed without major modifications. We could detect *Rickettsia* in 17.5% of the pools, including questing and feeding ticks. The obtained sequences comprised a near-complete genome, partial conjugative plasmid as well as *16S* rRNA, *ompA*, *sca1* and *gltA* gene segments (Table 2). The strain could be identified as *R. hoogstraalii* in two pools comprising feeding *Ha. parva* ticks, while the available data were insufficient for precise strain characterization in the remaining specimens. *Rickettsia* (order *Rickettsiales* genus *Rickettsia*) are intracellular Gram-negative bacteria that infect eukaryotic cells [30]. Several species are recognized, currently organized within distinct groups, according to the genome-wide sequence data [31]. *Rickettsia hoogstraalii* is closely related to *R. felis* and both strains are classified within the spotted fever group that includes species causing tick-borne infections in humans [32]. Despite *in vitro* cytopathic effects on various cell lines, the pathogenesis of *R. hoogstraalii* in vertebrate hosts remains unknown [32]. The isolation was accomplished from *Haemaphysalis sulcata* in Croatia and it has been detected in several tick species from various countries, including Cyprus, Ethiopia, Japan, Spain, the Indian Ocean islands and the USA [32–34]. *Rickettsia hoogstraalii* was initially identified in Turkey in 2014 in *Ha. parva* and the follow-up efforts have detected this strain in *Ha. parva* and *Haemaphysalis punctata* ticks in Central Anatolia [11, 35–37]. In addition to the near-complete genome with 98.3% identity to the prototype genome, we characterized a segment of the rickettsial plasmid and identified protein motifs with conjugative transfer functions. Despite their strictly intracellular life-cycle and reductive genomic evolution, several *Rickettsia* spp. have been shown to possess plasmids, with the possibility of horizontal, plasmid-mediated DNA exchange in ticks [38, 39].

We could not characterize the detected *Rickettsia* in five tick pools, despite the availability of relatively long *16S* rRNA gene sequences (Table 2). The *16S* gene is highly conserved among *Rickettsia*, where the similarity level between two species exceeds 97.2% [31]. This constitutes an impediment for significant inferences of intragenus phylogeny and hampers strain identification, which can be overcome by sequencing citrate synthase or outer surface proteins and surface cell antigens [31, 40]. Such data for the tick pools in question could not be produced in this setting, due to the relatively limited number of target sequence reads obtained.

Ticks have been documented to harbor diverse bacterial strains engaged in facultative or obligate endosymbiotic interactions with their hosts [6, 7]. Many distinct genera of bacteria, including strains collectively named as CLE, FLE and *Rickettsia*-like endosymbionts, have been identified in ticks [41]. CLE and FLE were detected mostly in ticks, with varying infection rates in different species. CLE are ubiquitous, geographically widespread and detected in several tick species as well as in the spleen of wild mammals [7, 42, 43]. We characterized *16S* rRNA gene sequences of CLE in 40% of the screened tick pools, which were the most frequently detected bacteria in the study cohort (Table 2). We detected CLEs in feeding as well as hunting/questing ticks, and observed a differential phylogenetic clustering of sequences according to the tick species (Fig. 2). The genus *Coxiella* is genetically divergent, with at least four highly divergent clades recognized, and CLE hosted by ticks are present in all clades [41, 44]. Interestingly, the phylogenetic patterns indicate that the well-known human pathogen *Coxiella burnetii*, the etiological agent of Q fever, has evolved from a tick-associated *Coxiella* [44]. This was also observed in our analysis where *C. burnetii* shared a common ancestor with *Ixodes-* and *Haemaphysalis*-associated sequences and formed a distinct clade among *Coxiella* (Fig. 2).

We further detected FLE in our cohort, with an incidence of 22.5%, occurring in hunting/questing ticks. FLEs are considered as an obligate symbiont alternate to CLE in some tick species and are, like CLE, genetically related to their pathogenic counterpart: *Francisella tularensis*, the etiological agent of tularemia [7, 41]. FLEs are widely distributed in Europe and identified in various tick species [45]. Interestingly, we observed a preferential detection of FLE in *Hyalomma* ticks (Table 1). Moreover, the FLE sequences formed phylogenetically distinct clusters associated with their tick hosts, suggesting differential evolutionary patterns in various hosts and ecological niches (Fig. 3). All FLE-related sequences remained distinct from pathogenic *Francisella*.

NGS provided *Borrelia 16S* rRNA gene sequences in 5% of the pools comprising hunting *H. aegyptium* ticks (Table 1). These sequences were identical to the previously characterized *Borrelia turcica* isolated from the same tick species [46, 47]. *Borrelia turcica* and closely related bacteria (*Borrelia* sp. tAG) are divergent from species associated with Lyme disease and relapsing fever, forming a third phylogenetic lineage within the genus *Borrelia* [48, 49], as observed in our analysis (Fig. 4). Also called reptile-associated *Borrelia*, members of this lineage are widely distributed, infecting various tick species [48–51]. Detected only in ticks or blood collected from tortoises so far, the consequences of human or animal exposure by these *Borrelia* are currently unknown [48, 49]. However, given the detection of several zoonotic agents and sporadic feeding on humans of *H. aegyptium* ticks, human infection by *Borrelia turcica* seems possible [51]. Therefore, characterization of the infecting strain in symptomatic individuals may provide information on the pathogenic potential of the members of this *Borrelia* lineage.

The outcomes of vertebrate infections with bacterial endosymbionts or apparently non-pathogenic bacteria in ticks remain obscure. No information regarding FLEs and *Borrelia turcica* pathogenicity is currently available. However, mild human infections caused by *Coxiella*-like bacteria were documented [52] as well as asymptomatic equine and severe avian pet infections [53–55]. These findings suggest that occasional human infections may occur. Therefore, they should be investigated in tick-associated infections in humans and animals without detectable pathogens. Another aspect is that non-pathogenic bacteria may interfere in the replication of tick-borne pathogens, influencing their abundance in vectors and transmission to vertebrate hosts. We could identify CLE and *Babesia* co-infection in a single questing *R. sanguineus* complex specimen (P23; Table 1, Additional file 1: Table S1), which indicates that co-infections are not extremely rare and can be detected by using appropriate methods in field-collected ticks.

The NGS-based approach further provided protozoan *18S* rRNA gene sequences in 10% of the tick pools where *Babesia*, *Theilera* and *Hemolivia* spp. were identified (Table 1). The microorganisms could be characterized as *B. ovis* and *He. mauritanica* in *R. bursa* and *H. aegyptium* pools, respectively by pairwise comparisons and inferred phylogenies (Figs. 5 and 6). Babesiosis is prevalent in Turkey and *B. ovis*, the etiological agent of sheep babesiosis, was previously identified in *R. bursa* and *Rhipicephalus turanicus* ticks [35, 56, 57]. In addition to *B. ovis*, several other species were reported as well as a proposed novel *Babesia* in ticks and goats [9, 11, 58, 59]. Despite reliable identification of *B. ovis*, the *Babesia* sequence in the *R. sanguineus* complex pool (P23) remained unidentified due to insufficient sequence data.

The major shortcoming of this study is the relatively low number of total and target sequences obtained from tick pools. NGS-based approaches, when optimized for DNA/RNA deep sequencing, can produce up to 10^9^ reads [60], which surpasses the overall sequencing efficiency observed in this study. The lack of tick-associated microorganisms in 30% of the pools can be attributed to this particular limitation, along with the overabundance of background signals from the host. Several steps and factors within the NGS workflow may affect sequencing efficiency and depth, producing potential biases in the representation of the original sequences [61]. Our strategy involved utilization of standardized specimen processing and library preparation, comparable to PCR-based pathogen screening. Targeted amplification and NGS of the bacterial and protozoan rRNA or pathogenic microorganisms can be alternate strategies, such as those we previously developed for viral hemorrhagic fever agents [62]. For a detailed investigation of the tick microbiome, individual ticks should be evaluated with a deeper sequencing strategy which we plan to employ for co-infected specimens in upcoming studies.

## Conclusions

Using an NGS-based approach, we detected bacteria of the genera *Rickettsia*, *Coxiella*, *Francisella*, *Borrelia* and protozoans of the genera *Babesia*, *Theileria* and *Hemolivia* in questing and feeding ticks. A near-complete genome and the conjugative plasmid of *R. hoogstraalii* were assembled, along with several coding and non-coding *Rickettsia* genes in tick pools. Moreover, CLE and FLEs with the hosting tick species were documented.

## Additional files

Additional file 1: **Table S1.** Tick pools evaluated using high throughput sequencing. **Table S2.** Microbial detection rates in pools according to tick species. (XLS 38 kb)

Additional file 2: Near-complete *R. hoogstraalii* genome sequence, assembled from the *Ha. parva* pool P4. The sequence is available in FASTA format and observed gaps following alignment to the *R. hoogstraalii* strain Croatica genome (CCXM01000001) are indicated. (TXT 1410 kb)

Additional file 3: Alignment of the partial conjugative plasmid sequences of *R. hoogstraalii* p4 characterized in this study (GenBank: MH649269), with *R. hoogstraalii* strain Croatica (CCXM01000002), *R. felis* strain (CP000054) and *R. australis* strain Cutlack (CP003339). (PDF 63 kb)

## Abbreviations

CLE: *Coxiella*-like endosymbionts
FLE: *Francisella*-like endosymbionts
NGS: Next-generation sequencing
rRNA: ribosomal RNA
