# Supplementary material for: A cross-sectional screening by next-generation sequencing reveals Rickettsia, Coxiella, Francisella, Borrelia, Babesia, Theileria and Hemolivia species in ticks from Anatolia
Source: Parasit Vectors. 2019 Jan 11;12:26. doi: 10.1186/s13071-018-3277-7 (PMC6329055; doi:10.1186/s13071-018-3277-7)
Supplement: Supplementary file 3 — Alignment of the partial conjugative plasmid sequences of R. hoogstraalii p4 characterized in this study (GenBank: MH649269), with R. hoogstraalii strain Croatica (CCXM01000002), R. felis strain (CP000054) and R. australis strain Cutlack (CP003339). (PDF 63 kb) [file 13071_2018_3277_MOESM3_ESM.pdf]

```
                               10        20        30        40        50        60        70        80        90       100
                      ....|....|....|....|....|....|....|....|....|....|....|....|....|....|....|....|....|....|....|....|
RhoogstraaliiP4Turkey TTCTCATATACAAAGAAGCAGGGGACAAAATGCTGTTGCTACCGCTGCGTATATTTCTGCATCAAAATTAGTGTATAAAACCATAGATCCAGAGAGTGGC
RhoogstraaliiCroatica ..........................................T.....................................................C...
RfelisURRWXCal2       ...G................A....................AA.....A...........TAGT........A........T.CCTG..AT..A.C...T
RaustralisCutlack     --------............AA............A.......A........C...G.A..TAGT.................T..T...AA...A......

                              110       120       130       140       150       160       170       180       190       200
                      ....|....|....|....|....|....|....|....|....|....|....|....|....|....|....|....|....|....|....|....|
RhoogstraaliiP4Turkey GAAAAGATAAGCATTACTTATGATTTTACTAAAAAGCAAGGAGTAGTATTTAGCAAGATATTTGTACCTGAGGGGTTTGAAGATGTAGTATGGTTACAAG
RhoogstraaliiCroatica ....................................................................................................
RfelisURRWXCal2       ..GG.A.....T..A..C.T.........AC.............GA...A...T...........G.....A..C........C.CT.C....C......
RaustralisCutlack     ...GC...T..T........................A.......GAC..A...T...........G.....A..C..A..........CC...C.G....

                              210       220       230       240       250       260       270       280       290       300
                      ....|....|....|....|....|....|....|....|....|....|....|....|....|....|....|....|....|....|....|....|
RhoogstraaliiP4Turkey ATAGAGAGCAGTTATGGAACGTAGCTGAAGTACGAGAAAAAAGAGAAAATTCTAGAACTGCAGCACAAATAGAATTCTCATTACCAAAAGAGGTAAGTCA
RhoogstraaliiCroatica ................................................C.G.................................................
RfelisURRWXCal2       .......A.............G...A....C.A......-.....CGG.......G......T..G..........T...C.....CGT..A......A.
RaustralisCutlack     ....................ACG..A....C.A......C...GA.TG..G..........T.A.A....TTGT..TG.CC.....CGT.....G...G.

                              310       320       330       340       350       360       370       380       390       400
                      ....|....|....|....|....|....|....|....|....|....|....|....|....|....|....|....|....|....|....|....|
RhoogstraaliiP4Turkey GGCAGAAAATATTAGGCTAGTGGAGGAATATGTACAGAAAAACATAGTATCAAGAGGTATAGTTTGTGATGTAAATATACACTACGATAATCCTGATAAC
RhoogstraaliiCroatica ....................................................................................................
RfelisURRWXCal2       ..A.CT.........TT.G..A..AA.......GA.TG.GT.T.....CG.......AG.T..A..............T..T.................T
RaustralisCutlack     ..A.A........GAAT....A..A..G......A.C..G..TC.....G..C..........A...........................GTA......

                              410       420       430       440       450       460       470       480       490       500
                      ....|....|....|....|....|....|....|....|....|....|....|....|....|....|....|....|....|....|....|....|
RhoogstraaliiP4Turkey CCACATGTACATATTCAATATTTAACAAGAAGATTAGAGCAATTAATTAACAATGAAGTAGTGCTTGCAAAAACAAAGGACAGAGATTTAGAAAGTACAA
RhoogstraaliiCroatica ........................................G...........................................................
RfelisURRWXCal2       ..........................T....A........G....GAG..TGGC........A....G.........AA.T.....A...........G.
RaustralisCutlack     ..G.........T.C..G.T.............C......GT...GAC..TGG......GA.A..C.G...G.TT..A.CA......A..A...CC..T.

                              510       520       530       540       550       560       570       580       590       600
                      ....|....|....|....|....|....|....|....|....|....|....|....|....|....|....|....|....|....|....|....|
RhoogstraaliiP4Turkey AGTTTTTATATTTTGCAAGGGATCAGTGTGAGATAGAAATCAACAAAGTATATGAGGAAGTAGGGTTACCTTTTAGGGTAACCGCTAAGTCATTCAAAGA
RhoogstraaliiCroatica ....................................................................................................
RfelisURRWXCal2       ..............TTGC....CAG...C...............................C.........A.A...A.....A...........TG..AC
RaustralisCutlack     ..GCAG.G...AA.TTT...A.G..A......G.C...............................................T.................

                              610       620       630       640       650       660       670       680       690       700
                      ....|....|....|....|....|....|....|....|....|....|....|....|....|....|....|....|....|....|....|....|
RhoogstraaliiP4Turkey ACTTGGATTAGATCAAGAGCCAACGCATCATAAAGGGCCTGCTCATTATATGAAGGCAACTGAGCTTGAGGCAAAGAACAAAGAAATAATAGCCGAGAAT
RhoogstraaliiCroatica ....................................................................................................
RfelisURRWXCal2       G......A........ATA....A..T.....T...A..A......C.......CCT..............A.....A........G..C.....T......
RaustralisCutlack     G..G...C........A.T..G....G.........A....................T....................TC...C.......ATT......
```

```
                                   710       720       730       740       750       760       770       780       790       800
                         ....|....|....|....|....|....|....|....|....|....|....|....|....|....|....|....|....|....|....|....|
RhoogstraaliiP4Turkey    TCAAAGAAGATATATGAGAATCCGGAAATAGTATTTGGTCGCATTTCATATACGAAGCCTGTGTTTACTAAAGAGGATATAGTGATAGCATTATCAGATG
RhoogstraaliiCroatica    ....................................................................................................
RfelisURRWXCal2          G..................G...T.......................G..C.....A..A..............A......CT............T..C.
RaustralisCutlack        G...G............A.....A..G..............T............C...................A......CT............G...A

                                   810       820       830       840       850       860       870       880       890       900
                         ....|....|....|....|....|....|....|....|....|....|....|....|....|....|....|....|....|....|....|....|
RhoogstraaliiP4Turkey    CTTTAATGGCGCATTTAGTATCAAAAGGCAGCAATATTATTCAGGAACAAGAGGCTATTAGAGCATTTGGAAAAGGATTAGTTCAGGAGCAAGATAGTAG
RhoogstraaliiCroatica    ....................................................................................................
RfelisURRWXCal2          ..C......T..................A..TG.....GCAA.A..G.......................T..G..C.....AA.T.......CAG..GA
RaustralisCutlack        ..C......T.........G.......AT..TG......C.A..C.G...A.A...........C.....C.GG..G.....G.....A....G......

                                   910       920       930       940       950       960       970       980       990      1000
                         ....|....|....|....|....|....|....|....|....|....|....|....|....|....|....|....|....|....|....|....|
RhoogstraaliiP4Turkey    TGAAGATGCACCAAAAAGCGTAAG------------------------------------------------TAATAATCCAGCAGCAAGTGCAGATAAC
RhoogstraaliiCroatica    ........................------------------------------------------------............................
RfelisURRWXCal2          ..G.A..A..GA.C...ATA..GTGAATGATGCAGTAGGAACTGCGGCAGGAGGTGGAGCAGAGAATACAGA..G..GC.A..T.AA.GAGA.CAC....
RaustralisCutlack        .A........G..C...ATA...AGAATAATTCAGTCGTAATGGCTGC------------------------....G.C.A.....AC...AGCAC...T

                                  1010      1020      1030      1040      1050      1060      1070      1080      1090      1100
                         ....|....|....|....|....|....|....|....|....|....|....|....|....|....|....|....|....|....|....|....|
RhoogstraaliiP4Turkey    AACCCGGCTATCGAGCATCTGAACAAAGAATATGGAGAGGAGTTTTTAAGGCTTTATAATCAGCTGCTTGTATCAGACAAAATAGAGCTGATGGATCAGA
RhoogstraaliiCroatica    ..........................G.........................................................................
RfelisURRWXCal2          .....A............T.A.....G........C............C.......C........A........T.....G......T............
RaustralisCutlack        .....A............T.A.....G...........T........................T.A..G.....T..TC.G..T..T.............

                                  1110      1120      1130      1140      1150      1160      1170      1180      1190      1200
                         ....|....|....|....|....|....|....|....|....|....|....|....|....|....|....|....|....|....|....|....|
RhoogstraaliiP4Turkey    AAGATTTGATGGGTAGAATGCTATACTCCTTGAAATCAAGAGTGAATTTAGAGCATCGCTATGGTATCTGCTATAGAGGAGTTAAATGAAAAAGAAGAGC
RhoogstraaliiCroatica    ....................................................................................................
RfelisURRWXCal2          .............A....C......T...C.A...........A...C....A.G...T....-.......................ACT....GC..A.
RaustralisCutlack        .............A....CT..G..T...........T.....A........A.G...T....-.G......................CT..G.C..CA.

                                  1210      1220      1230      1240      1250      1260      1270      1280      1290      1300
                         ....|....|....|....|....|....|....|....|....|....|....|....|....|....|....|....|....|....|....|....|
RhoogstraaliiP4Turkey    ATAATCTTAATATTTTAGATAGCACTATTGGAGAAAGAAATAGTCTCAAAGAACAAATAGGCGATATGGTAAATGTAATAGGAGCAAACCTACAAAATAA
RhoogstraaliiCroatica    ....................................................................................................
RfelisURRWXCal2          ....G..G.............A............G.....C.....T..G...........T...T.A.........G......................
RaustralisCutlack        ....G.............................T...........T...............A.AT.A...T.....G..........A.....G.....

                                  1310      1320      1330      1340      1350      1360      1370      1380      1390      1400
                         ....|....|....|....|....|....|....|....|....|....|....|....|....|....|....|....|....|....|....|....|
RhoogstraaliiP4Turkey    GTTTAATGATAAGATAGGAGGTAGTTTAGGTTTAAAGCTTAATGTATTTGGCAGCAAAACTCATGAGTTTAGTACTGAGCAGATAGAAGCGATCTTATCG
RhoogstraaliiCroatica    ....................................................................................................
RfelisURRWXCal2          .............C....CA..G.......C..............T...........G.AG....CA......GA......A..CA....A...C.C..A
RaustralisCutlack        ..............................C............A.............G...A............G.........T...............
```

```
                                   1410      1420      1430      1440      1450      1460      1470      1480      1490      1500
                               ....|....|....|....|....|....|....|....|....|....|....|....|....|....|....|....|....|....|....|....|
RhoogstraaliiP4Turkey          GTCTGTAACGGCTCTGATATTAGCGTATTAGAGGGTAATCCTGGAAGCTGGTAAGACTTTCGTGCTGCGAGAGATAGTACGGCAGTACAAGAGGGCAGGA
RhoogstraaliiCroatica          ....................................................................................................
RfelisURRWXCal2                .......GT..............T...C.T..A..A.....C...-..............T...A..AA.........G..T........AGC.......
RaustralisCutlack              ..................G....T...C.T..A..C.........-T...T.............A..A............AT..A.....AGCA..G...

                                   1510      1520      1530      1540      1550      1560      1570      1580      1590      1600
                               ....|....|....|....|....|....|....|....|....|....|....|....|....|....|....|....|....|....|....|....|
RhoogstraaliiP4Turkey          TTTACAGTATTAGGAGCAGGACCAAGTAGCATATCTGCAAAGGTACTTAGTCGAAATGCCGGCATTAAAGCTGATAATACGAGTTTGCTACGTAAGAAAC
RhoogstraaliiCroatica          ..................................................................................------------------
RfelisURRWXCal2                ....AG...G....TA.T...........TG.......T.....TT.A.....T.....T..A.....G..A...........................A
RaustralisCutlack              ....AG..GG....TA.T..T.................T.....CT.A.....T.....T...........A...........C..A.......C....A

                                   1610      1620      1630      1640      1650      1660      1670      1680      1690      1700
                               ....|....|....|....|....|....|....|....|....|....|....|....|....|....|....|....|....|....|....|....|
RhoogstraaliiP4Turkey          TCACTGAGTCTAAAGGGGGAGATTTCAAGATAGATTTAAGCAGTAAATATTATGAGGAGGAGGAATAT-TTAAATCTATCGGTTGTGATTCGATCTTTAA
RhoogstraaliiCroatica          -----------.G..AA.CGA..............................................-...............................
RfelisURRWXCal2                ..GTA.....C.....A..C..C..T.................C........................T.A........T..............T..G..
RaustralisCutlack              .AGAA..A..A.G...A.CGA....T...C..........T.......................G...T.A........T.........G.TG....C.G

                                   1710      1720      1730      1740      1750      1760      1770      1780      1790      1800
                               ....|....|....|....|....|....|....|....|....|....|....|....|....|....|....|....|....|....|....|....|
RhoogstraaliiP4Turkey          CTTTATGCGATTC-GATGTGCTTGATAGTAAGACCGTACTGATTGTGGATGAGGCAAGCATGATAGAGCTTGCTAATATGGACTATCTATCCCACGAAGT
RhoogstraaliiCroatica          .........-.CAA.................A....................................................................
RfelisURRWXCal2                ....T.A..-...T.....A..............T...T....C..T.................T........................G.T.....G..
RaustralisCutlack              .GGC.A..C-.GAG..A..C..G.....C....G............A...A.......T...........................T..G..T.T.....

                                   1810      1820      1830      1840      1850      1860      1870      1880      1890      1900
                               ....|....|....|....|....|....|....|....|....|....|....|....|....|....|....|....|....|....|....|....|
RhoogstraaliiP4Turkey          ATTAAGAGCTAAGGCAAAGCTGGTGCTTGTTGGAGATAACAACCAGTTCACGGCTGTTGGTATGACTGGAGCATTTAACAAAGCTCGTAAGATTGCAGGA
RhoogstraaliiCroatica          ....................................................................................................
RfelisURRWXCal2                C.....GT.............A..A.......................T..A....................G...........A...............
RaustralisCutlack              T.....GT....A..T.....A..A.......................T..A.....A..........................C........A.....G

                                   1910      1920      1930      1940      1950      1960      1970      1980      1990      2000
                               ....|....|....|....|....|....|....|....|....|....|....|....|....|....|....|....|....|....|....|....|
RhoogstraaliiP4Turkey          GGCGTTAAATTATCAGAAGTTAGAAGACAGAAGAGATTAGAGTACAGACAGGCTACCGAAGCGATGGGACGCTTTGAGATGTAGGAAGCAATTGAGATAT
RhoogstraaliiCroatica          ...........................................................................T........................
RfelisURRWXCal2                ..A.....GC.TA.T.....A.........G.A...G..........G.....G..GAG...T.............CC...C.......C..........
RaustralisCutlack              T..T.C..GC.TA.TA.G............G.A.ACA.....C....GA.A..C..T.....T.............CA...ATT..C..C..........

                                   2010      2020      2030      2040      2050      2060      2070      2080      2090      2100
                               ....|....|....|....|....|....|....|....|....|....|....|....|....|....|....|....|....|....|....|....|
RhoogstraaliiP4Turkey          GCAGAAATTTAGGTGTTTTTAATATTAAGGATACTGAGGAAGAGGCTAAAAGCAGTTTAGTGTCATCATTTGCTAAAGAATATACTGAGCAGATGGAGAG
RhoogstraaliiCroatica          ....................................................................................................
RfelisURRWXCal2                AT.....A...AC...A.....C..........A....C.G..A.......CT..C.....TA.G.............................A..T.A
RaustralisCutlack              A......A....A.A.............T....A...A.............CT..C.....TG.....................................
```

```
                                 2110      2120      2130      2140      2150      2160      2170      2180      2190      2200
                       ....|....|....|....|....|....|....|....|....|....|....|....|....|....|....|....|....|....|....|....|
RhoogstraaliiP4Turkey  TCTTAAGCGAGATGATTTAATAGCTATGCGAAGTATTGCAATTGGAGCATACACGAACGAGAAGGTAGCTGAGTTTAACGTGCGAGTTCGTGAGGAATTA
RhoogstraaliiCroatica  ....................................................................................................
RfelisURRWXCal2        .......A..............................G..TA.......A...............................T............................---....ACAA....AA.AAGT..GC..
RaustralisCutlack      ...........................TA..........................T..G..T.....T................A......AGCAAG..AA.ATC...G...

                                 2210      2220      2230      2240      2250      2260      2270      2280      2290      2300
                       ....|....|....|....|....|....|....|....|....|....|....|....|....|....|....|....|....|....|....|....|
RhoogstraaliiP4Turkey  AAGCATAGTGGAGCGTTAAAGGGACAGGAAGTACTAGTTAGCAGCGGCGTGCGATTACTGCCGCTGATGAAAGGAGATCAAATAGTATTTGAAGAGAATA
RhoogstraaliiCroatica  ................................................AG..................................................
RfelisURRWXCal2        ........C.....A.........ACT.......A.A.......T....G....A.G...T.............C..C.G....................
RaustralisCutlack      ........A...........................A....T..T....GTA..A.G..A..............C.................C.......

                                 2310      2320      2330      2340      2350      2360      2370      2380      2390      2400
                       ....|....|....|....|....|....|....|....|....|....|....|....|....|....|....|....|....|....|....|....|
RhoogstraaliiP4Turkey  GTTTAAGATACGGTATTAGTAACGGAGAAGTGGGGACAATATTATCAGTTAAACCTTCTGTTAAATCGTTTAGTGGTAGTGCGAATAGTAAAGGTGAGAG
RhoogstraaliiCroatica  .........................G......................................................................G...
RfelisURRWXCal2        .............G........T........A..A..............C..G.....G..................G......G...............
RaustralisCutlack      ......................T..T.....T...........G.....C..G.....G.................C...A.A........T........

                                 2410      2420
                       ....|....|....|....|....|.
RhoogstraaliiP4Turkey  CGATGGCGATGGAATATTAAAAATAT
RhoogstraaliiCroatica  ..........................
RfelisURRWXCal2        T........C...T..C.T.......
RaustralisCutlack      .......A.C..TT..C.T.......
```
